# Clinical Characteristics and Molecular Subtyping of *Vibrio vulnificus* Illnesses, Israel

**DOI:** 10.3201/eid1412.080499

**Published:** 2008-12

**Authors:** Ronit Zaidenstein, Chantal Sadik, Larisa Lerner, Lea Valinsky, June Kopelowitz, Ruth Yishai, Vered Agmon, Michele Parsons, Cheryl Bopp, Miriam Weinberger

**Affiliations:** Assaf Harofeh Medical Center, Zerifin, Israel (R. Zaidenstein, M. Weinberger); Israel Ministry of Health, Jerusalem, Israel (C. Sadik, L. Lerner, L. Valinsky, J. Kopelowitz, R. Yishai, V. Agmon); Centers for Disease Control and Prevention, Atlanta, Georgia, USA (M. Parsons, C. Bopp); Tel Aviv University, Ramat Aviv, Israel (M. Weinberger)

**Keywords:** Vibrio infections, epidemiology, microbiology, prevention, molecular biology, Israel, tilapia, carp, perspective

## Abstract

During 1996–1997, a new *Vibrio vulnificus* biotype 3, which caused severe soft tissue infection after fishbone injury, emerged in Israel. We conducted a follow-up study from 1998 through 2005 to assess changing trends, outcomes, and molecular relatedness of the implicated strains. A total of 132 cases (71% confirmed and 29% suspected) of *V. vulnificus* biotype 3 infection were found. Most infections (95%) were related to percutaneous fish exposure, mainly tilapia (83%) or common carp (13%). Bacteremia, altered immune status, and history of ischemic heart disease were identified as independent risk factors for death, which reached a prevalence of 7.6%. Pulsed-field gel electrophoresis patterns of strains from 1998 through 2005 and from 1996 through 1997 showed a high degree of homogeneity and were distinct from those of *V. vulnificus* biotype 1. Infections caused by *V. vulnificus* biotype 3 continue affect the public’s health in Israel.

## Abstract 2

The genetically distinct biotype 3 has penetrated Israeli freshwaters and is causing severe illness in persons who handle tilapia or carp.

*Vibrio vulnificus,* a gram-negative bacterium of the family *Vibrionaceae*, is a worldwide inhabitant of salt water (*1**–**3*). *V. vulnificus* biotypes 1 and 2 are capable of causing severe human infection, including necrotizing fasciitis and septicemia; the death rate is substantial (*4**–**6*). Persons with chronic liver disease, particularly liver cirrhosis, are more prone to developing infection and at greatest risk for an adverse outcome (*7**,**8*). Other predisposing factors are iron overload and hemochromatosis and immunosuppression caused by steroid treatment, malignancy, HIV infection, renal failure, and organ transplantation (*1**,**9**,**10*).

During 1996–1997, a new biotype, *V. vulnificus* biotype 3, emerged as a cause of severe soft tissue infection and bacteremia in Israel (*11**,**12*). Several important features differentiate the illness caused by the new *V. vulnificus* biotype from previously described *V. vulnificus* infections. First, a new vector, a pond fish (tilapia) grown in fresh water, has been associated with *V. vulnificus* infection. Second, infection is caused by direct injury from the fish backbone while purchasing, cleaning, or handling the live fish, as opposed to contamination of a prior injury by immersion in seawater or ingestion of contaminated seafood. Bacteriologically, the new *V. vulnificus* biotype differs from other *V. vulnificus* strains by its biochemical features (salicin-, cellobiose-, citrate-, and lactose-negative, plus delayed reaction for o-nitrophenyl-β-d-galactopyranoside [ONPG]). These biochemical differences initially prevented correct identification of the strain by routine laboratory methods (*11**,**12*). Furthermore, molecular analyses using several methods have shown that *V. vulnificus* biotype 3 is genetically distinct from biotypes 1 and 2 (*11**–**15*). Bisharat et al. suggested that biotype 3 is a recombinant clone that may have emerged as a result of hybridization of 2 *V. vulnificus* populations (*14*). Currently, biotype 3 is geographically restricted to Israel; biotypes 1 and 2 have a worldwide distribution (*16*).

The 1996–1997 Israeli cluster involved 62 persons, with a slight male predominance (58%) and a median age of 56 years. Although no deaths were reported, 41 persons (66%) had conditions that required surgical debridement, 1 had total limb amputation, and 7 had finger amputations (*11*). A new, aggressive, live-fish marketing initiative in the northern part of Israel was implicated in the outbreak, and the outbreak was followed by new instructions from the Israeli Ministry of Health that recommended selling only precleaned, ice-chilled tilapia (*17*).

We studied the epidemiology and the trends in illnesses associated with the new *V. vulnificus* biotype 3 during a 7-year period (1998–2005) following the initial 1996–1997 cluster. Our study assesses the effects of infection, risk factors for death, possible spread to other fish species, and molecular relatedness of the *V. vulnificus* biotype 3 strain.

## Methods

### Clinical Data

Clinical data were obtained from the records of the Infectious Diseases Department, Epidemiology Unit, Israel Ministry of Health, Jerusalem. Subdistrict health offices reported data collected from persons with *V. vulnificus* infections; a standardized questionnaire was used. Investigation was initiated after passive reporting from primary physicians who treated patients with suspected *V. vulnificus* infection or when clinical isolates were positively identified as *V. vulnificus* biotype 3 by the Vibrio Reference Laboratory in the Government Central Laboratories, Israel Ministry of Health, Jerusalem. The data collected were patient’s age, sex, and place of residence; underlying diseases; circumstances of exposure and type of fish involved; time lapse from exposure to seeking treatment in the emergency department; site of infection; length of hospitalization; clinical symptoms; source of isolation; antimicrobial drug treatment; and outcome.

Cases were classified as laboratory-confirmed when a patient with suggestive history had *V. vulnificus* or *V. vulnificus* biotype 3 isolated from blood or soft tissue or as suspected when a patient had suggestive history without positive cultures. Suggestive history was considered to be the development of soft tissue infection or septicemia after recent (within 7 days) fish exposure or immersion in a water pool.

### Source of Isolates and Laboratory Diagnosis

Initial identification of *V. vulnificus* was performed in the microbiology laboratories of hospitals where infected patients had been admitted, using the API 20E strip (bioMérieux, Marcy-l’Etoile, France). The laboratories submitted the isolates for confirmation and further identification to the Vibrio Reference Laboratory, Government Central Laboratories in Jerusalem. Identification of *V. vulnificus* biotype 3 was performed solely in this laboratory by using biochemical tests (failure to ferment citrate, lactose, salicin, cellobiose, and a negative test result for ONPG).

**Figure 1:**
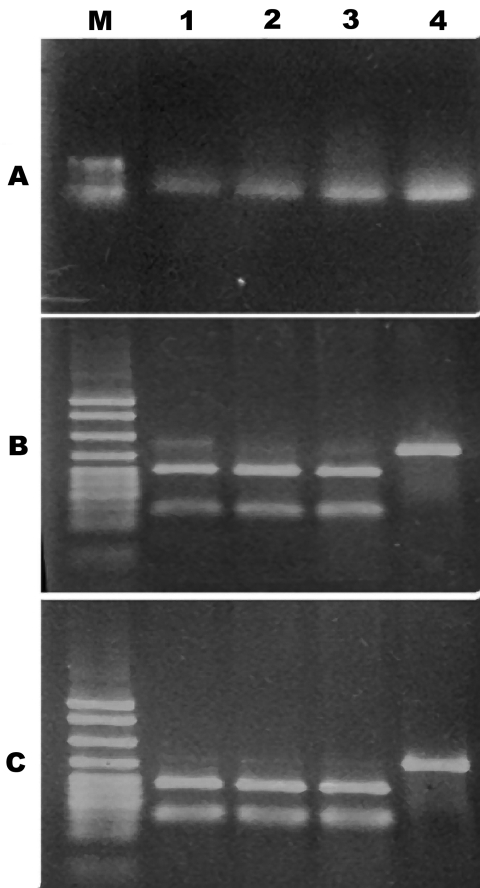
PCR restriction fragment length polymorphism of *Vibrio vulnificus* cytotoxin gene *vvhA*. A) PCR amplicon of *vvhA* gene restriction digested with B) *Pst*I or C) *Kpn*I. Gel shows molecular size standards (M) and *V. vulnificus* biotype 3 (lanes 1–3) and biotype 1 (lane 4).

Random isolates were also submitted for molecular analysis of the cytotoxin-hemolysin gene. This test was developed in the Vibrio Reference Laboratory and can differentiate between *V. vulnificus* biotype 3 and biotypes 1 and 2 by demonstrating the unique restriction fragment length polymorphism (RFLP) patterns of biotype 3 (Figure 1). Briefly, the *V. vulnificus* cytotoxin-hemolysin gene (*vvh*A) is amplified from crude bacteria lysate (boiling a loop full of bacteria suspended in 100 μL Tris-EDTA buffer for 10 min) using primers with the sequences RRCTH 5′-CAGCTCCAGCCGTTAACCGAACCACCCGC-3′ and LCTH 5′-TTCCAACTTCAAACCGAACTATGAC-3′. This step is followed by RFLP analysis. For the enzymatic restriction reaction, 2 μL of the PCR-amplified DNA was added to a reaction mixture to give a final volume of 20 μL, according to the manufacturer’s instructions (New England Biolabs, Ipswich, MA, USA), in 2 separate reactions. The restricted DNA was separated by electrophoresis in a 2% gel that was stained with ethidium bromide and visualized for the specific biotype 3 restriction patterns. Restriction sites for 2 enzymes, *Kpn*I and *Pst*I, existed in the sequence of *vvh*A of *V.* *vulnificus* biotype 3 but not in the corresponding *vvh*A gene of biotypes 1 and 2 (Figure 1).

### Molecular Subtyping

Twenty randomly selected laboratory-confirmed isolates of *V. vulnificus* biotype 3 from study years (1998–2003) plus 4 retrospective isolates from 1997 were sent to Centers for Disease Control and Prevention (CDC), Atlanta, Georgia, USA, for molecular subtyping. The isolates were recovered from the blood or wound sites of patients with a history of exposure to various fish. An additional isolate was recovered from the remnants of a tilapia fish found in the refrigerator of 1 of the infected patients (the fish remnants were diced and inoculated onto *Vibrio*-selective agar with and without enrichment). These 25 isolates were available for further molecular studies. Eight submitted human *V. vulnificus* biotype 1 isolates stored at CDC were used for comparison.

*V. vulnificus* isolates were subtyped by pulsed-field gel electrophoresis (PFGE) in accordance with the PulseNet protocol for *V*. *cholerae* (*18*) using *Sfi*I for primary enzyme digestion with 1 modification: thiourea (Sigma-Aldrich, St. Louis, MO, USA) was routinely added to the electrophoresis running buffer at a final concentration of 50 μmol/L (*19*) to prevent DNA degradation, which was commonly found during the initial runs. All DNA fingerprints were captured using a Gel Doc EQ system (Bio-Rad, Hercules, CA, USA). The PFGE fingerprints were analyzed in BioNumerics, version 4.0 (Applied Maths, Sint-Martens-Latem, Belgium). Gels were normalized by aligning the bands of the PulseNet universal standard *Salmonella* *enterica* serotype Braenderup (H9812) placed in every fifth lane on the gels (*20*). Dendrograms were made of the similarities of the DNA fingerprints by using the Dice similarity coefficient and unweighted pair group method with averages (unweighted pair group method with arithmetic mean) clustering. An optimization of 1.5% and tolerance window of 1.5% were used. The 25 isolates sent to CDC as described above were submitted for susceptibility tests using the E-test method according to Clinical Laboratory Standards Institute standards for *Enterobacteriaceae* (*21*).

### Statistical Methods

Proportions were compared by using the Fisher exact test or the χ^2^ test, and continuous variables were compared by using the Kruskal-Wallis test. Variables associated with death at the 0.05 significance level were entered into a stepwise forward logistic regression model for mortality rate. Analyses were performed by using SPSS version 15 software (SPSS, Inc., Chicago, IL, USA).

## Results

**Figure 2:**
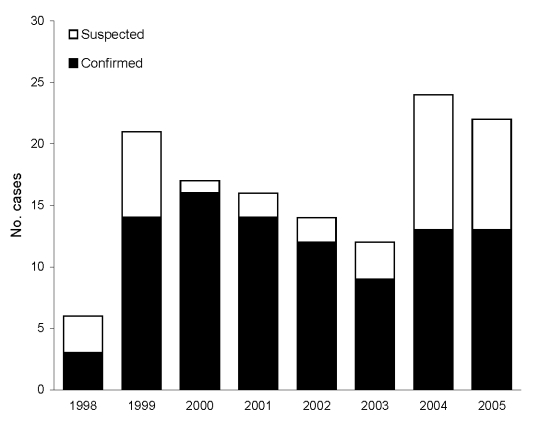
Annual distribution of laboratory-confirmed and suspected *Vibrio vulnificus* biotype 3 infections.

**Figure 3:**
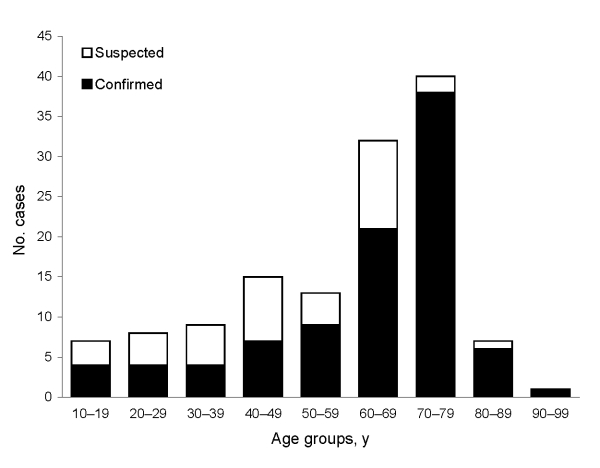
Age distribution of patients with laboratory-confirmed and suspected *Vibrio vulnificus* biotype 3 infections.

A total of 134 cases of *V. vulnificus* infection were identified during the 8-year study period from 1998 through 2005 (Figure 2). Most cases (96, 71.6%) were laboratory-confirmed; 70 (52%) were submitted to the Vibrio Reference Laboratory and identified as *V. vulnificus* biotype 3. PCR-RFLP analysis performed on 34 of these 70 isolates (49%) showed the unique *Kpn*I, *Pst*I pattern. Two patients with laboratory-confirmed *V. vulnificus* biotype 3 infection were excluded from further analyses because clinical data were missing. The median age of the remaining 132 patients was 66 years (mean 58.9 years, range 10–93 years) (Figure 3). Overall, infection rates for women were only slightly higher than those for men (1.1:1); this predominance increased in those >70 years of age (1.7:1). The sites of *V. vulnificus* isolation included wounds in 61 patients (65% of 94 patients with laboratory-confirmed infection), blood in 24 (26%), or both in 7 (7%). The source was not recorded in 2 patients.

Clinical characteristics of the patients and outcomes are summarized in Table 1. Compared to patients with laboratory-confirmed infection, patients with suspected infection were more likely to be men, with a longer incubation period after exposure, less severe clinical symptoms, a shorter hospitalization time, and a more favorable outcome. Information regarding the type of exposure was available for 93 patients. Most of these patients (75, 81%) were injured while purchasing or preparing fish for cooking. Fish were purchased from fish stores (52, 56%), mobile selling units (9, 10%), or stands near fishponds (14, 15%). Other types of exposures included involvement in fish marketing (14, 15%), either in selling (11/14) or cleaning the fishponds (3/14). One person was infected after fishing at a fishpond. Four persons had no connection to the pond fish industry. Three persons with suspected infection became ill after fishing in the Sea of Galilee, and 1 with laboratory-confirmed *V. vulnificus* biotype 3 infection reported immersion in a natural spring near Jerusalem with an open wound. Most of the injuries affected the hands; only 9 affected the legs.

**Table 1:** Clinical characteristics of patients with *Vibrio vulnificus* biotype 3 infections, Israel, 1998–2005*

Clinical characteristics	All patients	Patients with laboratory-confirmed infection	Patients with suspected infection	p value†
No. patients studied (%)	132 (100)	94 (100)	38 (100)	
Males, no. (%)	64 (48.5)	40 (42.6)	24 (63.2)	0.036
M:F ratio	0.94:1	0.7:1	1.7:1	
Mean age, y; median (range)	58.9; 66 (10–93)	63.1; 68 (10–93)	48.7; 47.5 (11–88)	NS
Clinical presentation				
Mean incubation time, h; median (range)	17.7; 12 (1–96)	13.9; 12 (0.5–48)	28; 20 (1–96)	<0.001
Bacteremia, no. (%)	31 (23.5)	31 (33.0)	NA	
Septic shock, no. (%)	11 (8.7)‡	10 (11.0)§	1 (2.9)¶	NS
Necrotizing fasciitis, no. (%)	16 (12.7)‡	16 (17.6)§	0	0.006
Uncomplicated cellulitis, no. (%)	92 (69.7)‡	62 (68.1)§	30 (85.7)¶	0.072
Abscess formation, no. (%)	12 (9.8)‡	7 (7.7)§	5 (14.3)¶	NS
Peritonitis, no. (%)	1 (0.8)‡	1 (1.1)§	0	NS
Underlying diseases#				
None, no. (%)	59 (44.7)	49 (52.1)	10 (26.3)	0.007
Liver disease, no. (%)	18(13.6)	18 (19.1)	0	0.002
Diabetes mellitus, no. (%)	18 (13.6)	13 (13.8)	5 (13.2)	NS
Ischemic heart disease, no. (%)	9 (6.8)	8 (8.5)	1 (2.6)	NS
Altered immune status,** no. (%)	13 (9.8)	9 (9.6)	4 (10.5)	NS
Hemolytic anemia, no. (%)	3 (2.3)	2 (2.1)	1 (2.6)	NS
Outcome				
Mean hospitalization time, d; median (range)	10.9; 9 (2–50)	12.7; 11 (2–50)	6.2; 5 (2–20)	0.003
Amputation, no. (%)	9 (6.8)	9 (9.6)	0	0.059
Death, no. (%)	10 (7.6)	10 (10.6)	0	0.062
No. patients with known fish exposure††	104	72	32	
Tilapia, no. (%)	86 (82.7)	60 (83.3)	26 (81.3)	NS
Carp, no. (%)	14 (13.5)	10 (13.9)	4 (12.5)	NS
Tilapia plus carp, no. (%)	2 (1.9)	1 (1.4)	1 (3.1)	NS
Other, no. (%)	2 (1.9)	1 (1.4)	1 (3.1)	NS

*NA, not available; NS, not significant. †For comparison between patients with laboratory-confirmed and suspected infections. ‡n = 126. §n = 91. ¶n = 35. #Patients may have >1 underlying diseases. **Due to malignancy or immunosuppressive state. ††Percentages of exposure to each fish type based on total no. patients with known fish exposure in each category.

**Figure 4:**
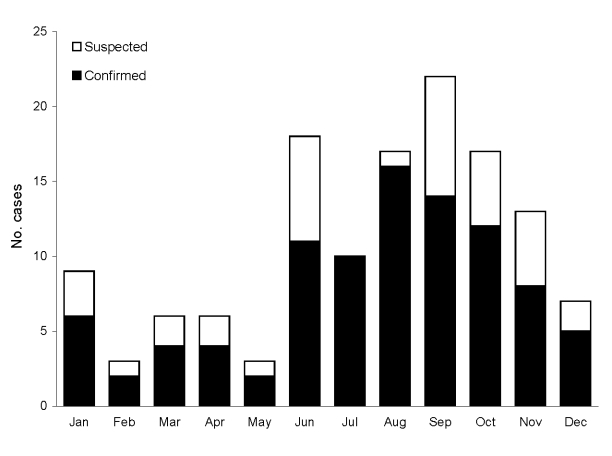
Seasonality of *Vibrio vulnificus* biotype 3 illnesses, Israel, 1998–2005.

Most of the infections (74%) occurred in the warm months of the year between June and November (Figure 4). Most patients resided in northern Israel, where freshwater fish aquaculture takes place. Only 6 patients lived in other areas of Israel (3 in the central part, 2 in the southern part, and 1 near Jerusalem). A total of 104 patients provided details regarding fish exposure. Tilapia (St. Peter’s fish) was the primary fish involved in 86 of these cases (83%), common carp (*Cyprinius carpio*) in 14 cases (13%), and both in 2 cases (2%). Two other patients reported exposure to saltwater fish: gilt-head sea brim (*Sparus aurata*) that was purchased in a fish store (laboratory-confirmed infection) and common gray mullet (*Mugil cephalous*) that was purchased at a fish stand near a fishpond (suspected infection).

### Course and Outcome

A total of 124 patients (94%) were hospitalized for a mean duration of 10.9 days (median 9 days, range 2–50 days). Information regarding treatment with antimicrobial drugs was available for 112 patients and included a variety of regimens. The most frequent regimens among these patients included only a single drug, usually ampicillin-clavulanate (47 patients, 42%), doxycycline (27, 24%), and ceftriaxone (6, 5%). Combinations of drugs were also used and included third-generation cephalosporins (ceftriaxone or ceftazidime) plus doxycycline (18, 16%), third-generation cephalosporins plus ampicillin-clavulanate (3, 3%), or doxycycline plus ampicillin-clavulanate (8, 7%).

Ten patients (7.5%) died and 9 (6.8%) underwent amputation of fingers or part of the arm (7 patients) or a leg (2). Two of the patients who underwent amputation subsequently died. Seven of the patients who died (70%) were >70 years of age, 2 were in their 50s, and 1 was in his 60s. All patients who died or underwent amputation had laboratory-confirmed infection.

Ninety patients with laboratory-confirmed infections were included in the analyses of risk factors associated with death. Factors that were significantly associated with death by a univariate analysis are outlined in Table 2. We could not show that any of the antimicrobial drug regimens influenced outcomes. Variables associated with death were entered into a stepwise forward logistic regression model. All initially entered variables remained in the model as independent predictors of a fatal outcome: presence of bacteremia (odds ratio [OR] 6.03, 95% confidence interval [CI] 1.2–30.8, p = 0.031), altered immune status (OR 6.7, 95% CI 1.1–40.8, p = 0.038), and history of ischemic heart disease (OR 15, 95% CI 2.5–91.1, p = 0.003) (p value for the model <0.001). When septic shock was added, only septic shock (OR 24.4, 95% CI 4.3–140.2, p<0.001) and history of ischemic heart disease (OR 9.7, 95% CI 1.3–72.7, p = 0.027) remained in the model (p value for the model <0.001). The model did not change when adjusted for age.

**Table 2:** Variables associated with death in laboratory-confirmed *Vibrio vulnificus* infection, by univariable analysis, Israel, 1998–2005*

Clinical characteristics	Alive,† n = 84	Dead,† n = 10	p value
Mean age ± SD, y	62.3 ± 18.2	69.2 ± 15.8	NS
Female, no. (%)	60 (49.2)	8 (80)	NS
Septic shock, no. (%)	41 (4.9)†	6 (60)	<0.001
Bacteremia, no. (%)	24 (28.6)	7 (70)	0.013
Ischemic heart disease, no. (%)	4 (4.8)	4 (40)	0.004
Altered immune status,‡ no. (%)	6 (7.1)	3 (30)	0.052

*NS, not significant. †n = 81. ‡Due to malignancy or immunosuppressive state.

### Antimicrobial Drug Susceptibility Tests

*V. vulnificus* isolates were susceptible to all antimicrobial agents tested by criteria for *Enterobacteriaceae*. The MIC ranges for ampicillin were 0.5–1.0 μg/mL; cephalothin, 4–8 μg/mL; chloramphenicol, 0.5–0.75 μg/mL; ciprofloxacin, 0.012–0.023 μg/mL; kanamycin, 4–16 μg/mL; nalidixic acid, 0.25–1 μg/mL; streptomycin, 8–16 μg/mL; tetracycline, 0.5–7.5 μg/mL; and trimethoprim-sulfamethoxazole, 0.064–0.094 μg/mL.

### Molecular Subtyping

**Figure 5:**
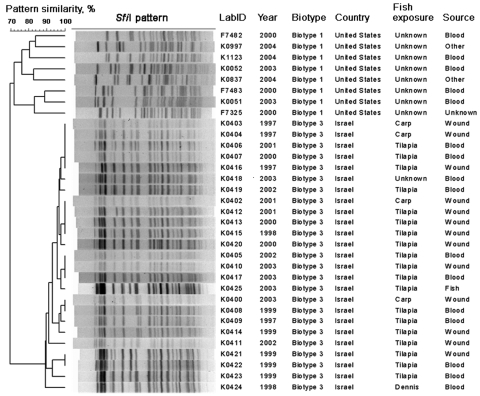
Dendogram comparing pulsed-field gel electrophoresis patterns of 25 *Vibrio vulnificus* biotype 3 isolates and a reference set of biotype 1 isolates when restricted with *Sfi*I enzyme.

Analysis of the PFGE subtyping results from 25 isolates (21 isolates from 1998 through 2003, 4 isolates from 1997) showed that the selected isolates represented 18 unique but similar (>88% pattern similarity; 1–3 fragment differences) PFGE fingerprint patterns regardless of the type of fish exposure (tilapia vs. carp) and year of isolation (Figure 5). A tilapia isolate from 2003 generated an *Sfi*I PFGE pattern, which was indistinguishable from the PFGE patterns of 3 patient isolates (1 from 2002, 2 from 2003) with reported exposure to tilapia from wound sites. When compared with biotype 1 strains reported from the United States, the biotypes separated into 2 distinct clusters with ≈70% pattern similarity (Figure 5).

## Discussion

Our study showed that infections caused by *V. vulnificus* biotype 3 continued to occur after the initial cluster during 1996–1997, with an average of ≈16 cases annually. Although the annual rate of infection during 1998–2005 was half the rate of infection during 1996–1997 (62 cases) (*11*), outcomes were more grave. Our study found that 10 persons (7.6%) died; no deaths were reported during the 1996–1997 outbreak (*11*). Possible explanations for this disparity could be that patients in our study were older (median age 66 vs. 56 years, respectively) with a higher proportion of laboratory-confirmed infections (70.1% vs. 53%, respectively). We have shown that patients without laboratory confirmation had a much milder form of the disease and a more favorable outcome.

Notably, our findings show a high proportion of infected women (52%), including 8 (80%) of the 10 patients who died. Previous studies have stressed a male predominance (*7**,**9**,**10**,**22**–**25*), and have even argued that female sex hormones protect against contracting the disease (*26*). With *V. vulnificus* biotype 3 infections in Israel, it was more likely that women purchased and prepared the fish before cooking, and thus were more likely to be exposed to fishbone injuries.

There are 3 major clinical syndromes of *V. vulnificus* illnesses, including primary bacteremia (mostly related to raw seafood consumption), wound infection (mostly related to immersion in contaminated water or to injury by seafood preparation), and gastroenteritis (after consumption of seafood or swallowing contaminated water) (*9**,**23**,**27*). Patients with primary bacteremia caused by non–biotype 3 strains are more likely to have predisposing conditions, particularly liver diseases in >80% of patients (*7**–**9*), whereas patients with wound infection are more likely to be previously healthy and have a more favorable outcome (*9**,**10**,**23**,**24**,**28**–**30*). The death rate may exceed 50% in the most seriously ill patients (*7**–**9**,**23*).

Our study describes a large, uniform group of patients who acquired infection through percutaneous exposure. All sought treatment for a wound infection, which in 18% was complicated by secondary bacteremia. In 38 patients (29%), laboratory confirmation of infection was lacking. These patients tended to delay seeking treatment and to have a milder form of infection, compared with patients who had laboratory-confirmed infection. Some of the patients who lacked laboratory confirmation may not have had *V. vulnificus* infection. However, patients with milder forms of *V. vulnificus* infection may have been less likely to undergo extensive microbiologic workup and the yield of cultures, if taken, would have been lower.

Only 45% of the patients in the group had underlying diseases. Liver disease occurred in 14% of patients overall, and in 19% of patients with confirmed cases, but was not a statistically significant risk factor for death. The case-fatality rate was 7.6% for the entire study population and 10.6 % for patients with laboratory-confirmed infection. These characteristics concur with prior reports of wound infection syndrome caused by non–biotype 3 *V. vulnificus* (*9**,**10**,**23**,**24**,**27**–**30*).

Of concern is our finding that *V. vulnificus* infection was not limited to tilapia exposure. Fourteen persons (13%) reported exposure to the common carp, or to both tilapia and common carp (2 persons, 2%). Tilapia and common carp are co-cultivated in several fishponds in northern Israel, which may have resulted in cross-contamination. For those exposed to salt water fish (gilt-head sea brim and common gray mullet, 1 person each), contamination may have occurred at the place of purchase. *V. vulnificus* infection after exposure to the common carp caused the death of 1 patient and the amputation of a finger in another. One person infected after exposure to the gilt-head sea brim also died. Notably, orthopedic surgeons from hospitals in northern Israel have also pointed out severe soft tissue infections, including those caused by *V. vulnificus*, after fishbone injury from the common carp (*31*).

The *V. vulnificus* biotype 3 strains studied were uniformly susceptible to all tested antimicrobial agents. These results concur with those of similar studies of clinical and environmental *V. vulnificus* biotype 1 isolates from the United States (*32**,**33*) and Taiwan (*22*). Only a few isolates from Taiwan showed resistance to ceftazidime and moxalactam.

Molecular subtyping of the *V. vulnificus* biotype 3 strains by PFGE showed no specific association between fish species and PFGE pattern. The results indicate that the biotype 3 strains are homogeneous with limited heterogeneity between the isolates but cluster distinct from biotype 1 strains. PFGE has been shown (*34*) to offer sufficient discrimination when subtyping biotype 1 strains, but published findings that evaluate the utility of PFGE to sufficiently discriminate between biotype 3 strains are limited. Modifications in restriction sites may alter the number and size of DNA fragments, which define the PFGE pattern and result in observable true differences.

A high degree of homogeneity among the *V. vulnificus* biotype 3 strains and distinction from the biotypes 1 and 2 has been also observed by other authors applying various methods, including random amplified polymorphic DNA, (*13*), multilocus sequence typing (*14**,**16*), and analysis of variations in simple sequence repeat loci (*35*). Notably, the latter method was able to demonstrate small-scale variations among the biotype 3 strains (*35*). The PFGE results also support the conclusion that this biotype 3 is distinct from the other *V. vulnificus* biotypes. The high degree of homogeneity is another indicator that the emergence of biotype 3 is a recent evolutionary event (*14**,**16**,**36*).

We identified independent risk factors for death in our group, including bacteremia, altered immune status, and history of ischemic heart disease. Septic shock was also found to be a strong predictor of death; however, septic shock may also be an outcome variable. Nonetheless, prior reports have also identified septic shock or hypotension as important risk factors for death (*8**,**23*). A recent large study from Taiwan (*29*) found that treatment with third-generation cephalosporins combined with tetracycline was an independent predictor of lower death rates in a subgroup of patients with hemorrhagic bullous necrotic cutaneous lesions. We did not demonstrate any correlation between a specific antimicrobial drug regimen and death rate in our study population. The predisposing diseases that were associated with death in previous reports were liver disease and neutropenia (*8**,**23*). Ischemic heart disease was not previously recognized as a classic predisposing factor predicting death; however, in the *Vibrio*-associated wound infections after Hurricane Katrina in Louisiana, USA, ischemic heart disease occurred in 7 of 13 patients (54%) with more severe illness (*25*).

Before the introduction of *V. vulnificus* biotype 3 into the fish aquaculture no infections were reported in Israel (*37*). Also, no reports have been made of *V. vulnificus* infection acquired through marine activities in the Mediterranean Sea. Almost the entire impact of *V. vulnificus* infections in Israel is associated with the freshwater fish industry. In response to the new threat, the Israeli Ministry of Health has issued regulations forbidding the selling of live, uncleaned tilapia (*11**,**38*). Fish stores and fishpond workers have been instructed to use protective gloves when handling fish, to keep fresh fish packed in ice, and to prevent direct contact between buyers and live fish. The public has been instructed accordingly. Apparently, compliance with these regulations is not universal.

Our findings outline the substantial effects of *V. vulnificus* illnesses in Israel and support a call for more strict regulations of fresh fish marketing as well as public education. Research efforts should focus on how *V. vulnificus* has penetrated the freshwater aquaculture in Israel and ways in which this trend can be reversed.
